# Colchicine poisoning treated with hemoperfusion and hemodialysis: A case report

**DOI:** 10.1002/ccr3.6419

**Published:** 2022-10-07

**Authors:** Maral Ramezani, Babak Mostafazadeh, Mitra Rahimi, Peyman Erfan Talab Evini, Shahin Shadnia

**Affiliations:** ^1^ Department of Pharmacology, School of Medicine Arak University of Medical Sciences Arak Iran; ^2^ Traditional and Complementary Medicine Research Center Arak University of Medical Sciences Arak Iran; ^3^ Toxicological Research Center, Department of Clinical Toxicology, Loghman Hakim Hospital Shahid Beheshti University of Medical Sciences Tehran Iran

**Keywords:** colchicine, hemodialysis, hemoperfusion, poisoning

## Abstract

This case report described an improved case of colchicine poisoning using hemoperfusion and hemodialysis.

## Abstract 2

The purpose of this study is to show the effectiveness of hemodialysis and hemoperfusion in colchicine toxicity when the patient does not respond to other treatments or other methods cannot be applied due to the symptoms caused by poisoning.

## 1 INTRODUCTION

Colchicine is a medicine that can be extracted from plants such as *Colchicum autumnale* and *Gloriosa superba*. Its therapeutic application has been confirmed for familial Mediterranean fever (FMF), gout, amyloidosis, Behcet's syndrome, pericarditis, arthritis, pulmonary fibrosis, vasculitis, biliary cirrhosis, pseudo‐gout, and specific spondyloarthropathy.^1^, ^2^ The therapeutic index of colchicine is narrow and, therefore, the interval between non‐toxic, toxic and lethal doses is short.^3^ Colchicine poisoning has no specific antidote. Early detection of poisoning is important because exposure can lead to multiple organ failure and death.^3^ We describe a case of colchicine poisoning treated with hemoperfusion and hemodialysis, with recovery and hospital discharge.

## 2 CASE REPORT

A 36‐year‐old married woman was referred to Loghman Hakim Hospital in Tehran 2 h after taking 10 acetaminophens 500 and 20 colchicine 1 mg tablets. The patient had no history of suicide, psychiatric illness or substance abuse. She had no history of previous illness or liver disease. Colchicine was not a drug used by the patient herself. Vital signs were as follows: heart rate of 73 beats per minute, respiratory rate of 13 breaths per minute, and blood pressure was 110/70 mmHg. On examination, the pupils were symmetrical with an average size of 3–4 mm, examinations of the heart, lungs, skin, and intestinal sounds were normal, and the patient had no trauma, ulceration, or sweating. She had severe nausea and vomiting.

Preliminary laboratory test results included venous pH 7.48, PCO2 33 mmHg, HCO3 24.7 mEq/L, blood urea nitrogen 34 mg/dl, creatinine 0.8 mg/dl, serum sodium 140 mEq/L, serum potassium 3.7 mEq/L, blood glucose 138 mg/dl, white blood cell count 5.5 × 103/μl, hemoglobin 14.2 g/dl, platelet count 130 × 103/μl, Aspartate transaminase (AST) 33 U/L, Alanine transaminase (ALT) 24 U/L, Alkaline phosphatase (ALP) 152 U/L, Creatine Phosphokinase (CPK) 152 U/L, Lactate dehydrogenase (LDH) 390 U/L, Prothrombin time^2^ 13, Partial Thromboplastin Time (PTT) 13, International Normalized Ratio (INR) 1 and Acetaminophen concentration 4 h after ingestion was below 5 μg/dl (Table 1).

**TABLE 1:** Selected laboratory results of patient

	Upon arrival	11 h	14 h	23 h	35 h	41 h	50 h	59 h	74 h	83 h	89 h	98 h
PH	7.48	7.39		7.41	7.38	7.43	7.44	7.41	7.3	7.37	7.46	
PCO_2_ (mmHg)	33	40.3		40.3	41.2	41.9	39.3	44.6	46	47.4	36.6	
HCO_3_ (mEq/L)	24.7	24.9		26.1	24.6	27.9	26.6	28.5	23	28	26.3	
BUN (mg/dl)	34		23		20		18	15				33
Cr (mg/dl)	0.8		0.8		0.8	0.9	0.9	0.8				0.9
Na (mEq/L)	140		142		137		137	137		140		138
K (mEq/L)	3.7		3.7		3.2		3	3.2		3.3		3.2
WBC (10^3^/mm^3^)	5.5		4		6.7	5.9	5.8	5.4	5.6	6.3	6.3	
Hb (g/dl)	14.2		12.3		12.2	11.9	11.5	13.2	11.9	11.6	11.6	
Plt (10^3^/mm^3^)	130		124		64	64	77	73	71	93	93	
BS (mg/dl)	138											
AST (U/L)	33											
ALT (U/L)	24											
ALP (U/L)	152											
CPK (U/L)	59											
LDH (U/L)	390											
PT	13											
PTT	34											
INR	1											
β‐HCG	0.6											
APAP Concentration	<5											

Considering that the plasma level of acetaminophen and based on Rumack Matthew's nomogram, acetaminophen poisoning was ruled out and colchicine poisoning was mainly considered.

Serum dextrose saline (DS) 1 litter every 8 h, ondansetron 4 mg IV/State, pentazole 40 mg IV/State, cardiac monitoring and pulse oximetry, vitamin B6 and oxygen therapy with mask (if saturated oxygen percentage below 92) were administered. The patient had nausea and vomiting on the second day. To eliminate the drug, she was given a multi‐dose of active charcoal (MDAC) 25 g in 240 cc of water every 4 h orally and the absence of diarrhea, Sorbitol 70 g every 8 h orally were given. She had severe nausea and vomiting untreated with supportive care, so receiving activated charcoal and sorbitol did not work well. Finally, due to severe gastrointestinal symptoms, vomiting and lack of proper response to medical treatments, the patient became a candidate for hemodialysis‐hemoperfusion. Continuous hemodialysis for 8 h and hemoperfusion with Jeffron HA 330 cartridge for 8 h were performed in parallel. Arterial blood gases, white blood cell count, hemoglobin, platelet count and other factors were monitored continuously (Table 1).

On the third to fifth days of hospitalization, the patient was alert and able to eat and drink. Laboratory test results were normal except for serum potassium levels. The patient developed hypokalemia from the third day. On the fifth day, after psychiatric consultation, the patient was discharged in stable condition. The patient was followed up one and 2 weeks after intoxication. The results of laboratory test 1 week after intoxication showed that the patient's liver enzymes activity was elevated (Table 2). Livergol tablet, dry extract of milk thistle (Silybum marianum), was administered 140 mg every 8 h. In experiments 2 weeks after intoxication, liver enzymes activity has returned to normal (Table 2).

**TABLE 2:** Selected laboratory results 1 and 2 week after poisoning

	Bun	Cr	WBC	Hb	Plt	BS	AST	ALT	ALP
1 week after poisoning	22	0.9	6.1	12.9	113	99	42	118	226
2 weeks after poisoning			5.7	13.2	195		22	28	195

## 3 DISCUSSION

Colchicine has anti‐inflammatory properties. Colchicine can block metaphase due to its anti‐mitotic effects. The toxic effects of colchicine are probably due to its anti‐mitotic activity in proliferating tissues such as bone marrow.^4^, ^5^ The maximum recommended daily dose for colchicine is 1.8 mg per day.^1^ Colchicine is rapidly absorbed from the gastrointestinal tract. Serum concentrations peak at 0.5–3.0 h after ingestion.^3^, ^6^ It undergoes extensive hepatic first‐pass metabolism due to its systemic bioavailability (25% to 50%).^3^, ^6^, ^7^ Its protein binding is 10%–50% and volume of distribution ranges between 2 and 12 L/kg but reaches up to 21 L/kg in overdose. Colchicine and its metabolites undergo enterohepatic recirculation.^3^, ^6^, ^8^

Gastrointestinal and coagulation disorders were reported at doses below 0.5 mg/kg, bone marrow aplasia in patients taking 0.5–0.8 mg/kg, and death at doses more than 0.8 mg/kg in acute use.^3^, ^6^ The clinical course of acute colchicine poisoning may be divided into three phases. The first (gastrointestinal) phase reflects gastrointestinal mucosal damage (nausea and vomiting, abdominal distress, and diarrhea). The second (multi‐organ) phase is characterized by multi organ dysfunction and metabolic derangements are also common (acute kidney injury, sepsis, rhabdomyolysis, electrolyte imbalances & etc). The third phase, which is characterized by recovery of bone marrow depression with rebound leukocytosis, resolution of organ failure, and can be followed by complete recovery.^3^, ^6^, ^9^

Measurement of colchicine concentration in body fluids is not common and no consistent association with disease severity has been reported.^6^, ^9^ Although in some studies its concentration in the body has been measured.^10^, ^11^ Treatment of colchicine poisoning is supportive. Decontamination is done by gastric lavage and active charcoal. Because the drug and its metabolites have an enterohepatic cycle, multiple‐dose activated charcoal (MDAC) is also recommended.^3^, ^6^ Many studies have reported that hemodialysis and hemoperfusion are not effective for colchicine poisoning due to their high volume distribution.^3^, ^6^, ^8^ In Goldfrank's book, it is stated that due to the large volume of distribution of colchicine, the use of hemodialysis and hemoperfusion are not a viable options except in cases of kidney failure.^6^ In the article by Sadiq et al.,^8^ it is mentioned that dialysis does not remove colchicine and that patients undergoing dialysis need a dose reduction due to their impaired renal function. However, in various cases, hemodialysis and hemoperfusion have been used along with other methods to treat colchicine intoxication.

A total of 43 cases of colchicine poisoning in China have been reported that plasma exchange combined with continuous veno‐venous hemodialysis filtration can increase survival time.^12^ In one case, a 68‐year‐old woman underwent extracorporeal life support (ECLS) and hemodialysis to treat colchicine intoxication.^13^ Hemoperfusion was performed on a 19‐year‐old woman poisoned with colchicine to remove toxins from the blood.^14^

Our case was a case of poisoning with an oral dose of 20 mg colchicine, which was performed continuous hemodialysis for 8 h and hemoperfusion for 8 h in parallel. Gastrointestinal decontamination was performed for the patient with multiple doses of activated charcoal and sorbitol. The patient eventually recovered and was followed up for up to 2 weeks after intoxication. During treatment, the patient showed an increase in the activity of liver enzymes and hypokalemia, which was treated appropriately.

Colchicine overdose is an uncommon but life‐threatening complication that manifests itself with progressive multi‐organ dysfunction. Using extracorporeal removal methods such as hemodialysis and hemoperfusion to remove the drug from the blood, although rarely used, can be effective and save the patient.

## AUTHOR CONTRIBUTIONS

MR collected the data for the study and wrote the manuscript. SS and BM revised the manuscript for grammar and syntax mistakes. MR and PETE corrected the manuscript for its scientific basis and revised the manuscript for grammar and syntax mistakes.

## FUNDING INFORMATION

No additional funding for the execution of the present study was received.

## CONFLICT OF INTEREST

The authors declare that they have no competing or conflict of interests.

## CONSENT

Informed written consent was obtained from the patient to publish this case report. A copy of the written consent form is available for review by the journal editor.

## Data Availability

The data and materials used in the current study are available from the corresponding author on reasonable request.
